# 

**Published:** 1888-07-28

**Authors:** 


CONTENTS.

**The Bitter Cry of the General Practitioner** - 269

**The "Universal Review"** - 270

**Is a General Register for Nurses Desirable?** By Mr. Henry Bonham-Carter, Honorary Secretary the Nightingale Fund - 271

**The Doctor's Pulpit.**—The Waste of Brain Resources - 273

**The Student's Column** - 274

**The Metropolitan Hospital Sunday Fund, 1888.**—Awards and Criticisms - 275

**Notes and News** - 276

**Everybody's Page** - 278

**Annotations.**—The Extension of the Leeds Infirmary—Anti-Vaccination Tactics: The Borax Trick—Roses in the Rain - 279

**Physiology and Its Uses.**—The Bones of the Upper Limb - 280

**Turning Over New Leaves.**—A Socialist Novel—Indian Diseases—Abortive Treatment of Specific Febrile Disorders—The Teachers' Bible - 281

**An Ishmaelite.** By Leslie Keith, Author of "The Chilcotes," "East and West," etc. - 282

**Amusements with Profit for all Ages.**—For Men and Women.—Children's Corner.—Puzzles, etc. - 284

**EXTRA SUPPLEMENT.—THE NURSING MIRROR.**

En Passant.—The Middlesex Institute—Cured by Thermometer—Encounter with a Madman—Florence Nightingale's Works—Nursing in Germany—A School of Midwifery—The Magazines and Nursing - lxv

Hints for the Sick-room. By M. M. Basil, M.A. - lxvi

Original Articles.—Nurses in Novels - lxvi

Everybody's Opinion.—Saint William's Hospital - lxvii

Notes and Queries.—Exchange and Mart - lxvii

The Nurses' Bookshelf.—"Nurses and Nursing" - lxvii

Appointments.—Lectures.—Vacancies.—Examination Questions lxviii

**Hospitals urgently needing Annual Subscriptions, Donations, and Legacies.**

**METROPOLITAN HOSPITAL SUNDAY FUND.**

